# Impact of Psychiatric Comorbidity on Cognitive Performance and EEG Theta/Beta Ratio: A Preliminary Study

**DOI:** 10.3390/brainsci16010034

**Published:** 2025-12-25

**Authors:** Wendy Verónica Herrera-Morales, Karen Nicte-Ha Tuz-Castellanos, Julián Valeriano Reyes-López, Efraín Santiago-Rodríguez, Luis Núñez-Jaramillo

**Affiliations:** 1 Laboratorio de Neurofisiología, Departamento de Ciencias Médicas, División de Ciencias de la Salud, Universidad Autónoma del Estado de Quintana Roo, Chetumal CP 77039, Quintana Roo, Mexico; 2 División de Investigación y Posgrado, Facultad de Ingeniería, Universidad Autónoma de Querétaro, Cerro de las Campanas s/n, Santiago de Querétaro CP 76010, Querétaro, Mexico; 3 Unidad de Neurodiagnóstico “Dr. Moisés López”, Facultad de Química, Universidad Autónoma de Querétaro, Cerro de las Campanas S/N-Edificio 5, Centro Universitario, Santiago de Querétaro CP 76017, Querétaro, Mexico; 4 Psiquiatría Integral Juriquilla S.C. Acueducto de Zacatecas 516, Santiago de Querétaro CP 76230, Querétaro, Mexico; 5 Diagnóstico, Tratamiento e Investigación Neurológica, S.C. Prolongación Pino Suárez No. 357-A Col. Galindas, Santiago de Querétaro CP 76178, Querétaro, Mexico

**Keywords:** psychiatric comorbidities, cognitive performance, impulsiveness, theta/beta ratio, brain electrical activity

## Abstract

**Background/Objectives:** Psychiatric conditions are highly prevalent and among the leading causes of disability worldwide. Comorbidities are common in psychiatric patients but are not adequately addressed in diagnostic manuals such as the DSM-5. Understanding the impact of comorbidities on patients’ symptoms and brain activity could improve the personalization of therapeutic approaches, leading to better outcomes. Given the complexity of this task, a feasible strategy is to examine how comorbidities affect brain activity and a condition commonly observed in psychiatric patients, such as cognitive impairment. **Methods:** In this study, we assessed impulsiveness, working memory performance, and theta/beta ratio in controls and in subjects exhibiting symptoms of depression, ADHD, and suicide risk. Participants differed in the presence of alcohol use disorders, in addition to the aforementioned symptoms, either presenting no alcohol use disorder (DAS), hazardous alcohol consumption (DAS-H), or risk of alcohol dependence (DAS-D). **Results:** All three comorbid groups (DAS, DAS-H, DAS-D) showed increased impulsiveness compared with controls, while the DAS-D group also exhibited higher motor impulsiveness than both the DAS and DAS-H groups. A widespread increase in theta/beta ratio was observed only in the DAS group. **Conclusions:** These results indicate that comorbid alcohol use disorders modulate motor impulsiveness and theta/beta ratio in subjects with symptoms of depression, ADHD, and suicide risk. The findings underscore the importance of considering comorbidities when personalizing treatment strategies for psychiatric patients.

## 1. Introduction

Psychiatric disorders have a high prevalence worldwide, affecting about 970 million people, including children, adolescents, and adults [1], and thus impacting quality of life across the lifespan. Mental disorders represent one of the leading causes of disability worldwide, with personal, social, and economic consequences [2]. Given the public health relevance of these conditions, numerous studies have examined their neurophysiological substrates as well as potential therapeutic approaches to alleviate their symptoms.

It is common for patients with a psychiatric condition to present one or more comorbid psychiatric disorders, as has been reported for ADHD [3,4], alcohol use disorders [5,6,7,8] and depression [9,10,11]. However, studies conducted on psychiatric patients often address a single condition, occasionally two, but rarely three or more. Still, the presence of comorbidities in psychiatric patients is undeniable, making it difficult to investigate the specific effects of particular comorbidities on patient symptomatology. Similarly, studies on the neurophysiological correlates of psychiatric conditions predominantly focus on a single disorder. It should be noted that the impact of comorbidities is not fully captured by commonly used diagnostic manuals such as the Diagnostic and Statistical Manual of Mental Disorders (DSM-5) [12,13].

Previous research has consistently documented differences in brain activity stemming from the presence of a single comorbidity. For instance, patients diagnosed with ADHD have been reported to exhibit higher absolute theta power compared to patients with comorbid Autism Spectrum Disorder (ASD) and ADHD [14]. Another study comparing individuals with ADHD with or without comorbid Internet Gaming Disorder (IGD) found that those with ADHD and comorbid IGD presented lower relative delta and higher relative theta power in temporal regions than those with only ADHD. Furthermore, subjects with comorbid ADHD and IGD displayed higher intra-hemispheric coherence for the delta, theta, alpha, and beta bands between electrode locations P4 and O2, and for the theta band specifically between Fz and Cz, as well as between T4 and T6 [15]. In the context of substance use, a study focused on long-abstinent alcoholics, with or without comorbid Major Depressive Disorder (MDD), reported reduced P3b amplitudes compared to controls during a visual oddball task only when the MDD comorbidity was absent [16]. A subsequent investigation involving individuals with a history of, but not current, alcohol dependence and MDD found that this group showed greater reward-related delta activity during a monetary reward task compared to three distinct comparison groups: individuals with past-only alcoholism, individuals with past-only MDD, and healthy controls [17]. Interestingly, all participants in this latter study were currently free of symptoms, highlighting the enduring influence of past disorders on brain electrical activity. Regarding affective disorders, one study found that women with childhood-onset depression showed reduced left lateral frontal activity (indexed by alpha asymmetry) compared with women who had the same condition with comorbid anxiety and healthy controls. The authors suggest that, in their sample, comorbid anxiety may mask the prefrontal asymmetry typically observed in women with childhood-onset depression [18]. Similarly, a study in adolescents reported that the group with MDD and comorbid anxiety showed lower left frontal cortical activity than healthy controls, whereas the group with MDD alone did not differ significantly from either of the other two groups [19].

Understanding the impact of comorbidities on patients’ brain function and symptomatology could lead to improved treatment personalization, resulting in greater effectiveness and better quality of life for these patients. Given the complexity of this task, it is useful to examine the influence of comorbidities on a common feature shared by various psychiatric conditions, such as cognitive impairment. This approach allows for determining how comorbidities affect cognitive function and the associated changes in brain electrical activity.

Patients diagnosed with psychiatric disorders, while categorized based on specific criteria detailed in instruments like the DSM-5, frequently exhibit other non-core symptoms. One such symptom, commonly observed across various psychiatric conditions, is cognitive impairment. This impairment can affect a patient’s performance across a wide variety of skills requiring distinct cerebral functions and may manifest with different patterns of affectation depending on the specific psychiatric condition. Significant cognitive impairment has been consistently reported in patients with alcohol use disorders [20,21], depression [22,23], or ADHD [24,25].

Furthermore, because cognitive impairment is present across a wide range of psychiatric conditions, regardless of the specific symptoms observed in each, it represents a potential bridge for initiating a comprehensive study of psychiatric patients and their everyday reality. It is noteworthy that while in some cases, like ADHD, cognitive impairment is one of the main features of the disorder [24,25], there are other cases, such as depression, in which cognitive performance may or may not be part of the symptoms experienced by each patient, even though cognitive impairment is one of the main contributors to functional outcomes in depression. This often leads to an underestimation of cognitive function in patients with this disorder, as cognitive deficits (in executive function, memory, or attention) frequently persist even after the remission of depressive symptoms and can continue to affect daily life. This underscores the need for specific follow-up of cognitive symptoms in psychiatric patients, in addition to monitoring improvement in the diagnosed disorder [22,23].

Psychiatric disorders carry a substantial societal impact, rendering their diagnosis and treatment a significant challenge. The presence of comorbidities and the inherent heterogeneity of symptoms within a single disorder can critically influence both the diagnostic process and the efficacy of the chosen treatment [13]. Therefore, achieving a more profound understanding of how these factors influence brain function will be instrumental in refining diagnostic accuracy and optimizing treatment selection. Ultimately, this approach will contribute to a higher quality of life for patients.

The purpose of this work was to determine whether the presence of comorbid symptomatology influenced brain electrical activity and/or cognitive performance in subjects with various comorbid symptoms. Given that it is not possible to predict the precise combination of comorbidities present in a population, the approach used in this study was to assemble the groups according to the combinations of symptoms most frequently observed in the sample. In the present work, we examined cognitive performance and brain electrical activity in subjects with comorbid psychiatric symptomatology in an effort to determine whether this factor produces significant changes that could be relevant for patient treatment.

## 2. Materials and Methods

### 2.1. Participants

Health sciences students were recruited during their regularly scheduled classes early in the academic semester. Those who expressed interest in participating were subsequently provided with a comprehensive explanation of all study procedures and signed an informed consent form. All procedures were conducted in strict accordance with the Declaration of Helsinki, and the study protocol received official approval from the Committee for the Evaluation of Research Functions of the Health Sciences Division. Participants self-reported having no current or past neurological conditions and not taking any pharmacological treatments that could influence brain electrical activity; only those who met both criteria were included in the study.

Inclusion criteria for this study were being at least 18 years old, either male or female, not currently under pharmacological treatment for any condition or pregnant, and enrolled in the first or second year of their study program. Participants who did not complete all tests or whose EEG recordings did not allow quantitative analysis were excluded from the study. Participants were asked not to consume caffeine, alcohol, or any other substance that might affect brain activity during the 48 h prior to EEG recording. EEG recording and cognitive testing were carried out between 9 a.m. and 3 p.m.

### 2.2. Clinical Assessment of Psychiatric Symptoms

The presence of psychiatric symptoms was assessed using validated instruments, with interpretation and final evaluation conducted by a qualified psychiatrist. Specifically, ADHD symptoms were evaluated using the Spanish version of the Adult ADHD Self-Report Scale (ASRS-V1.1). The ASRS-V1.1 is an 18-item self-report questionnaire designed to screen for symptoms of Attention-Deficit/Hyperactivity Disorder (ADHD) in adults (aged 18 and older). This instrument is derived from the World Health Organization Composite International Diagnostic Interview (2001) and utilizes wording specifically tailored to reflect symptom manifestation in the adult population, ensuring consistency with both the DSM-IV and DSM-5-TR criteria [26]. This Spanish version of the instrument has been previously validated in Spanish-speaking populations [27,28] and specifically within the Mexican population [29].

To detect depressive symptoms, participants completed the Beck Depression Inventory (BDI-II) [30], The BDI-II is a self-report measure of symptomatology composed of 21 Likert-type items and has been previously validated in Spanish in the Mexican population [31,32].

Suicide risk was assessed using Plutchick’s test, a 15-item scale with dichotomous responses. It assesses previous suicide attempts, intensity of current suicidal ideation, feelings of depression and hopelessness, and related factors, and has been already tested in Mexican population [33,34].

Impulsiveness was assessed using the Barratt Impulsiveness Scale (BIS-11), a 30-item self-report questionnaire designed to measure the multifaceted construct of impulsivity. The items are grouped into three distinct impulsivity subscales: cognitive impulsivity, motor impulsivity, and non-planning impulsivity [35]. The specific version utilized in this study (BIS-11) has been previously validated in Spanish-speaking populations [36,37,38] and reported in the Mexican population [39]. Impulsiveness was included in this investigation as a crucial indicator of cognitive function because it reflects the efficiency of executive control in processes such as working memory and inhibition. Moreover, impulsivity modulates performance across various cognitive tasks, thereby representing a key behavioral manifestation of cognitive regulation [40,41].

Alcohol use disorders (alcohol dependence and hazardous alcohol consumption) were assessed using the Spanish version of the Alcohol Use Disorders Identification Test (AUDIT). The AUDIT is a screening questionnaire designed by the World Health Organization (WHO) to identify risky and harmful alcohol use, as well as potential dependence [42]. It consists of 10 standardized questions, whit a thematic structure, where the questions are designed to assess different dimensions of alcohol consumption over the past year. It consists of 10 standardized questions with a thematic structure, designed to assess different dimensions of alcohol consumption over the past year. The instrument is available in various languages (including Spanish) on the WHO website. We used its Spanish version, which has already been validated in both its paper [43,44,45] and online [46] formats in different countries, including Mexico.

### 2.3. Working Memory Test

Working memory was evaluated using the Sternberg Working Memory Test, implemented via the e-Prime 2.0 software, as previously reported by our research group [47] and others [48,49,50]. The assessment consisted of 48 trials. In each trial, participants were sequentially presented with a random series of one to six digits appearing one after another on the screen. Following the final digit of each series, the computer emitted an auditory cue (a beep sound), which was followed by the presentation of the test digit in red. Participants were instructed to determine whether the test digit was present or absent in the preceding sequence. The accuracy index and reaction time of each participant were recorded for subsequent statistical analysis.

### 2.4. Electroencephalogram (EEG) Recording

EEG was recorded in a dimly lit room, with participants comfortably seated and instructed to keep their eyes closed. Signals were acquired with 16-bit resolution at a sampling rate of 240 Hz using a 19-channel Medicid Fenix electroencephalograph (Neuronic Mexicana S.A. de C.V., Mexico City, Mexico). The low-frequency filter was set at 0.5 Hz, the high-frequency filter at 30 Hz, and a 50/60 Hz notch filter was applied. Nineteen electrodes were positioned according to the international 10–20 system at FP1, FP2, F7, F8, F3, F4, T3, T4, C3, C4, T5, T6, P3, P4, O1, O2, Fz, Cz, and Pz, mounted in an electrode cap (Electro-Cap International, Inc., Eaton, OH, USA). Linked mastoids served as the reference, and Fpz was used as ground. Resting-state EEG for qEEG analysis was obtained during continuous eyes-closed recording, outside any task-related stimulus presentation or response phases.

### 2.5. Visual Analysis of EEG

All EEG recordings were initially inspected visually by a board-certified clinical neurophysiologist (ES-R). Continuous resting-state EEG segments were reviewed in the time domain to confirm wakefulness and the presence of posterior alpha activity, and to identify potential artifacts. Epochs containing artifacts such as eye blinks, eye movements, muscle activity, electrode pops, movement artifacts, or clear signs of drowsiness (e.g., alpha attenuation accompanied by the appearance of theta slowing) were rigorously rejected. For subsequent quantitative analysis, artifact-free resting-state segments, each with a minimum length of 2.56 s, were manually selected until a total duration of at least 60 s was achieved for every participant. All selected segments corresponded exclusively to the eyes-closed resting condition and were not time-locked to any stimulus presentation or behavioral responses. The EEG signals were originally recorded utilizing linked mastoids as reference. Prior to spectral analysis, all data segments were re-referenced offline to the average reference, a standard procedure in quantitative EEG studies, to mitigate the influence of the initial reference montage on the resulting topographical power distributions.

### 2.6. Quantitative Analysis of EEG (qEEG)

Quantitative spectral analysis was performed using the Neuronic software package (Neuronic Mexicana S.A. de C.V., Mexico City, Mexico). The software applies fast Fourier transforms (FFT) to consecutive, non-overlapping time windows of 2.56 s. The duration of this window (T) determines the frequency resolution of the resulting spectrum according to Δf = 1/T, yielding a resolution of approximately 0.39 Hz. Thus, the power spectrum is sampled on a discrete frequency grid in 0.39 Hz steps. This window length reflects a compromise between achieving sufficiently fine spectral resolution and maintaining approximate stationarity of the EEG within each epoch.

Absolute power (AP) spectra were computed for each electrode and each artifact-free epoch and then averaged across epochs. Theta power was defined over 3.91–7.42 Hz and beta power over 12.89–19.14 Hz [51]. These numerical limits correspond to the FFT bins whose center frequencies fall within the conventional physiological ranges of theta (approximately 4–8 Hz) and low beta (approximately 13–20 Hz), given the 0.39 Hz frequency resolution. In other words, the apparently “non-round” limits (3.91–7.42 Hz and 12.89–19.14 Hz) arise directly from the discretization of the spectrum into 0.39 Hz bins and do not reflect a different physiological definition of the bands.

Although 2 s epochs are commonly used in resting-state qEEG studies, epoch length primarily determines FFT frequency resolution rather than the physiological definition of canonical bands. Accordingly, our use of 2.56 s epochs (Δf ≈ 0.39 Hz) instead of 2 s epochs (Δf = 0.50 Hz) does not alter the interpretation of theta and low-beta activity and does not limit comparability with studies reporting the conventional 4–8 Hz and 13–20 Hz ranges; it only affects the exact bin boundaries used to compute band-limited power.

We utilized absolute power (AP) rather than relative power measures, as our primary interest lay in the absolute magnitude of oscillatory activity within these specific frequency bands. Relative measures were avoided because they can potentially obscure global power changes when the total power exhibits substantial inter-individual variability. To mitigate non-physiological inter-individual variability, the Global Scale Factor (GSF), as implemented in the Neuronic software, was applied specifically to the theta and beta AP values [52]. The theta/beta ratio was then calculated for each derivation as Theta AP/Beta AP.

The selection of this particular EEG marker was based on its established relevance for studying cognitive performance in general [53,54], as well as its frequent and critical use in studies involving ADHD [55,56,57] which represents a common comorbidity across all experimental groups (excluding the control group).

### 2.7. Statistical Analysis

For the analysis of the theta/beta ratio, derivations were grouped into three regions: Frontal (F, including Fp1, Fp2, F3, F4, F7, F8 and Fz), Centro-parietal (CP, including C3, C4, P3, P4, Cz, and Pz), and Temporo-occipital (TO, including O1, O2, T3, T4, T5, and T6). The theta/beta ratio from the electrodes within each region was averaged, as previously reported by our group and others [58,59,60,61]

We used a mixed-design analysis of variance (Mixed ANOVA), with brain region, working memory scores or impulsiveness subtype as the within-subjects variable and comorbidities as the between-subjects variable, applying Greenhouse-Geisser corrections when necessary. Partial eta squared (η^2^p) was used to determine effect size. Separate mixed ANOVAs were conducted to assess the effect of comorbidities on theta/beta ratio, working memory and impulsiveness. When a significant group effect was found, paired comparisons were performed to identify specific inter-group differences, followed by ANOVA with Bonferroni post hoc tests to determine differences between groups across regions or impulsiveness styles. In all ANOVAs, *p*-values were adjusted for multiple comparisons using Bonferroni correction.

All analyses were conducted using SPSS software (version 18.0).

## 3. Results

### 3.1. Subjects

After excluding subjects who did not satisfy the inclusion criteria, failed to complete all assessments, or those whose EEG recordings were unsuitable for quantitative analysis, a total of 318 participants were included in the final study sample. The mean age of the sample was 19.471 ± 0.101 years, comprising 193 females and 125 males. Eighty-five participants scored negative for all assessed conditions, constituting the control group. The remaining 233 participants exhibited symptoms for one or more of the evaluated psychiatric conditions, categorized as follows: 68 rated positive for suicide risk, 79 rated positive for depression, 152 rated positive for ADHD, 105 rated positive for hazardous alcohol consumption, and 31 rated positive for alcohol dependence.

Based on the comorbidities presented by the participants, we assembled four groups. Subjects who tested positive for depression, ADHD, and suicide risk were included in the DAS group (n = 11; age 19.482 ± 0.44 years; 10 females, 1 male). Subjects who tested positive for depression, ADHD, suicide risk, and hazardous alcohol consumption were included in the DAS-H group (n = 18; age 19.385 ± 0.342 years; 10 females, 8 males), while those who tested positive for depression, ADHD, suicide risk, and alcohol dependence were included in the DAS-D group (n = 8; age 19.471 ± 0.101 years; 6 females, 2 males). Subjects who tested negative on all assessments were considered controls (CTRL). Since these participants greatly outnumbered the other groups, a random selection of controls was performed using SPSS software to ensure reliable statistical analysis. As a result, the CTRL group included 13 subjects (age 20.859 ± 1.109 years; 6 females, 7 males).

### 3.2. Impulsiveness

Mixed ANOVA revealed an effect of group (F(3,46) = 10.374, *p* < 0.0001, η^2^p = 0.403), an effect of impulsiveness subtype (F(2.026,93.201) = 542.162, *p* = 0.000, η^2^p = 0.922), and an interaction group x impulsiveness subtype (F(6.078,93.201) = 8.018, *p* = 0.000, η^2^p = 0.343. Pairwise comparison showed differences between CTRL and DAS (*p* < 0.01), DAS-D (*p* < 0.001), and DAS-H (*p* < 0.01) groups.

The ANOVA test revealed a significant effect of group for cognitive (F(3,46) = 5.42, *p* < 0.01, η^2^p = 0.261), non-planned (F(3,46) = 6.458, *p* < 0.01, η^2^p = 0.296), motor (F(3,46) = 12.463, *p* < 0.001, η^2^p = 0.448) and total (F(3,46) = 10.411, *p* < 0.001, η^2^p = 0.404) impulsiveness.

**Figure 1:**
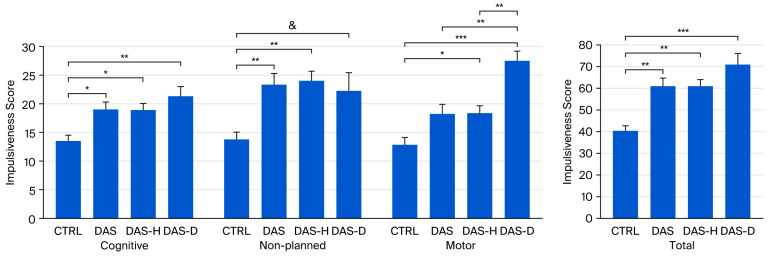
Impulsiveness scores for CTRL, DAS, DAS-H and DAS-D groups. Score ± SEM. * *p* < 0.05, ** *p* < 0.01, *** *p* < 0.001, & non-significant tendency (*p* < 0.1).

Post hoc tests conducted following the ANOVAs show that, for cognitive impulsiveness DAS (18.909 ± 1.468, *p* < 0.05), DAS-H (18.722 ± 1.209, *p* < 0.05) and DAS-D (21.25 ± 1.677, *p* < 0.01) groups presented higher scores than CTRL group (13.462 ± 1.136) (Figure 1).

Regarding non-planned impulsiveness, post hoc test revealed higher scores in the DAS (23.273 ± 1.996, *p* < 0.01) and DAS-H (23.994 ± 1.16, *p* < 0.01) groups when compared with the CTRL group (13.692 ± 1.273). Moreover, we found a non-significant tendency for a higher score in the DAS-D (22.125 ± 3.383, *p* < 0.1) when compared with controls (Figure 1).

For motor impulsiveness, post hoc analysis revealed higher scores in the DAS-H (18.278 ± 1.437, *p* < 0.05) and the DAS-D (27.5 ± 1.701, *p* < 0.001) groups when compared with the CTRL group (12.846 ± 1.224). Additionally, the DAS-D group presented a higher motor impulsiveness score than the DAS group (*p* < 0.01) and the DAS-H (*p* < 0.01) groups (Figure 1).

Bonferroni’s post hoc test also shows increased total impulsiveness scores in the DAS (60.636 ± 4.368, *p* < 0.01), the DAS-H (61 ± 3.314, *p* < 0.01) and the DAS-D (70.875 ± 5.488, *p* < 0.001) groups, compared to the CTRL group (40.231 ± 2.708) (Figure 1).

### 3.3. Working Memory Test

Mixed ANOVAs were conducted on the reaction time and accuracy index across the different conditions evaluated in the working memory test: when the test digit was present in the series (target trial), when the test digit was not present in the series (non-target trial), and when all trials were combined (overall performance). These analyses revealed no statistically significant differences between the studied groups in any of the parameters assessed.

### 3.4. Theta/Beta Ratio

Mixed ANOVA revealed an effect of group on theta/beta ratio (F(3,46) = 4.581, *p* < 0.01, η^2^p = 0.23), and an effect of region (F(2,92) = 27.451, *p* < 0.001, η^2^p = 0.37).

**Figure 2:**
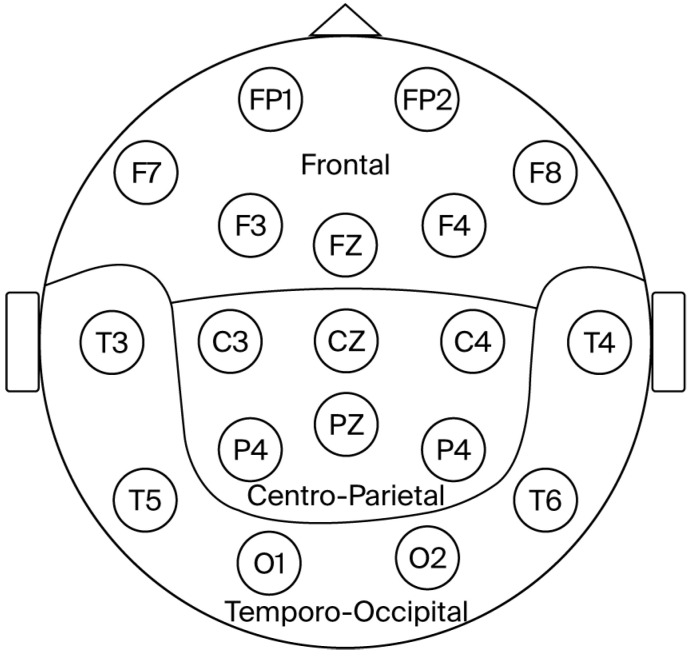
Diagram depicting the brain regions analyzed.

Regarding the effect of group at different brain regions (Figure 2), ANOVAs performed on theta/beta ratio revealed an effect of group at Frontal (F(3,46) = 4.35, *p* < 0.01, η^2^p = 0.822), Centro-parietal (F(3,46) = 3.9, *p* < 0.05, η^2^p = 0.203) and Temporo-occipital (F(3,46) = 4.541, *p* < 0.01, η^2^p = 0.228) regions.

Bonferroni’s post hoc tests revealed that the DAS group presented a higher theta/beta ratio than the DAS-H and the DAS-D at Frontal (DAS 3.912 ± 0.676; DAS-H 2.313 ± 0.199, *p* < 0.05; DAS-D 2.076 ± 0.299, *p* < 0.05) and Temporo-occipital (DAS 2.827 ± 0.47; DAS-H 1.634 ± 0.153, *p* < 0.01; DAS-D 1.679 ± 0.188, *p* < 0.05) regions, while DAS group presented higher theta/beta ratio than DAS-H group at Centro-parietal region (DAS 3.419 ± 0.739; DAS-H 1.733 ± 0.187, *p* < 0.05).

**Figure 3:**
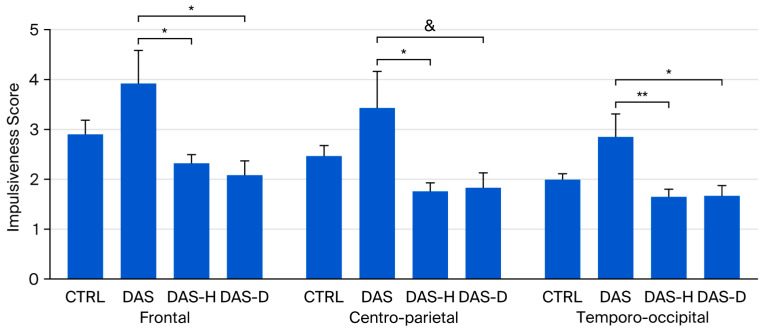
Theta/Beta ratio in CTRL, DAS, DAS-H and DAS-D groups af Frontal, Centro-Parietal and Temporo-occipital regions. Ratio ± SEM. * *p* < 0.05, ** *p* < 0.01, & non-significant tendency (*p* < 0.1).

Additionally, a non-significant tendency suggested higher theta/beta ratio in DAS group compared to DAS-D group at Temporo-occipital region (DAS-D 1.832 ± 0.313, *p* < 0.1) (Figure 3).

## 4. Discussion

In this study, we examined the effect of symptoms from different psychiatric conditions on cognitive performance (impulsiveness and working memory) and brain activity (theta/beta ratio) in these groups. The rationale behind our approach was to address a common situation in the general population—the presence of comorbidities—in order to determine whether they influence brain function. In this context, we found that comorbidities do affect both the neurophysiological correlates of symptoms and cognitive performance.

While the precise combination of symptoms addressed in our study may not fully reflect the most prevalent comorbidities observed in the general clinical population—defined as individuals seeking diagnosis and treatment for psychiatric conditions in clinical institutions—it does effectively address the main objective of this investigation. Our primary goal was to determine whether the presence of additional comorbidities produces a measurable difference in brain activity and cognitive performance among subjects presenting with a given set of symptoms. This question holds substantial relevance because pharmacological treatments are designed to modulate brain activity to alleviate symptoms, an effect that is highly sensitive to the surrounding neurochemical environment. Thus, if the presence of additional symptomatology modifies basal brain activity, and if this activity is intrinsically linked to neurotransmitter release, this modification could profoundly impact the efficacy and outcome of a chosen pharmacological treatment. Although our study is acknowledged as preliminary due to the modest sample sizes, it nonetheless permits the observation of changes in brain activity directly attributable to additional comorbidities within the specific symptom profiles defined across our study groups.

It should be noted that this study was not intended to determine the prevalence of the evaluated conditions in our population, but rather to characterize the neuropsychological and electrophysiological profile of subjects presenting specific combinations of psychiatric symptoms. This distinction is important because participation in the study was voluntary; only individuals who were interested responded to the invitation and visited our laboratory for more information about enrollment. Consequently, there may have been a bias toward higher participation among subjects who perceived themselves to be at risk for one or more of the studied conditions. Therefore, the prevalence of the symptomatology observed in this sample may differ from that reported in the general population, without affecting the relevance of this study for its intended purpose.

Regarding cognitive performance, our ANOVAs revealed no significant differences between the groups in working memory, encompassing both the accuracy index and reaction time. However, a closer inspection of the data indicated a large dispersion (high variance) across all groups for all measured working memory performance parameters. Consequently, this elevated variance led to the failure to achieve statistical significance. While this high dispersion is partially attributable to the modest sample size, it is notable that the variances for impulsiveness and the theta/beta ratio were sufficiently small to yield statistically significant differences between groups with relevant effect sizes, despite the limited number of subjects in each group (as illustrated in Figure 1 and Figure 3). This suggests that the higher dispersion observed in the working memory variables is also influenced by other factors. One possibility is that the combination of symptoms assessed does not necessarily result in a uniform change in cognitive performance specific to working memory. Given that impulsiveness did show variation consistent with the specific set of comorbid symptoms, our results collectively suggest that different aspects of cognitive function may be differentially affected by the presence of comorbidities. A definitive conclusion on this issue would require a more comprehensive battery of neuropsychological tests and larger group sizes.

In general, all groups with psychiatric conditions exhibited higher impulsiveness scores than controls, with the exception of non-planned impulsivity, where the DAS group showed only a non-significant trend toward higher scores compared to controls. Additionally, for motor impulsiveness, the DAS-D group scored markedly higher than the CTRL, DAS, and DAS-H groups (Figure 1). Impulsiveness is present across a wide variety of psychiatric disorders and is considered to influence performance on cognitive tests, as it reflects the efficiency of executive control [40,41]. A recent study analyzed the presence of different impulsiveness subtypes in various psychiatric conditions. Among the conditions examined, alcohol use disorders were associated with higher motor impulsiveness scores. However, that study did not distinguish between hazardous alcohol consumption and alcohol dependence, and the specific comorbidities present in our study were not addressed, making direct comparisons difficult [62].

While previous investigations have examined the influence of single comorbidities on brain electrical activity in patients diagnosed with ADHD [14,15], alcoholism [16,17] or depression [18,19], we found no existing study specifically addressing the presence of multiple, co-occurring comorbidities. The presence of symptoms for multiple conditions was, notably, not uncommon among our participants, who presented with the combinations reported here alongside others that were excluded from analysis due to the limited number of subjects with that precise symptomatic profile. A more comprehensive understanding of how these comorbidities interact to produce a given set of neurophysiological correlates remains beyond the scope of our current study, as such an endeavor would require substantially larger sample sizes and likely a more profound, in-depth analysis of brain activity data. Nevertheless, the preliminary results presented herein strongly underscore the importance of developing future studies specifically designed to gain a better understanding of the neurophysiological implications stemming from multiple comorbidities.

In our study, quantitative EEG analysis revealed a higher theta/beta ratio only in the group testing positive for suicide risk, depression, and ADHD, but not in the groups that additionally presented either alcohol dependence or hazardous alcohol consumption (Figure 3). The theta/beta ratio is typically elevated in individuals with cognitive impairment across different conditions [54,63,64], and has been proposed as an indicator of cognitive performance [53]. It is well documented that alcohol use disorders induce long-term changes in brain activity [65,66,67,68]. In our study, participants were instructed to abstain from caffeine, alcohol, or any other substance that might affect EEG activity, allowing us to observe the neurophysiological correlates of the conditions themselves rather than the acute effects of substance intake, so the effects observed are presumably due to long term effects of alcohol use disorders on brain activity. At first glance, our results may suggest that the presence of alcohol use disorders (either hazardous consumption or dependence) “reduces” the impact of the other conditions on the theta/beta ratio, as the DAS group alone exhibited higher theta/beta values (Figure 3), highlighting the influence of added comorbid symptoms on brain activity. This observation aligns with a previous study that reported reduced left lateral frontal activity in women with childhood-onset depression compared with healthy controls; however, this difference was no longer evident in women who also had comorbid anxiety [18].

A minor methodological limitation is that we used 2.56 s epochs instead of the more common 2 s epochs in resting-state qEEG studies. However, this choice only affects the exact numerical boundaries of the discrete spectral bins (0.39 Hz vs. 0.50 Hz resolution) and not the underlying theta (4–8 Hz) and low-beta (13–20 Hz) bands, nor the interpretability of the present results.

Subjects in our study presented with symptoms across various psychiatric conditions and were organized into four groups: DAS (Depression, Suicide Risk, and ADHD); DAS-H (Depression, Suicide Risk, ADHD, and Hazardous Alcohol Consumption); DAS-D (Depression, Suicide Risk, ADHD, and Alcohol Dependence); and Controls (CTRL). The specific combination of symptoms included in each experimental group was dictated by the frequency of these comorbidities among the enrolled participants. While this grouping strategy is somewhat unorthodox, it facilitates the study of a realistic clinical scenario where individuals often present symptoms of multiple psychiatric conditions, although it inherently complicates the interpretation of results. It is noteworthy that, with the exception of the control subjects, all participants shared a common core of symptoms (depression, suicide risk, and ADHD), differing primarily in the presence of alcohol use disorders (hazardous consumption, dependence, or absence of alcohol use disorder). Consequently, it is challenging to assess the specific contribution of each of the three core disorders to cognitive performance and brain electrical activity. However, this structure provides a consistent common reference point from which to evaluate the distinct effect of adding the symptomatology of another disorder. The purpose of this work is not to propose new clinical entities resulting from symptom combinations, but rather to explore how the alterations in brain function that underpin these symptoms influence cognitive performance and brain activity. Our findings suggest that the presence of comorbidities does indeed imply a difference in both cognitive performance (impulsiveness) and brain activity (theta/beta ratio). Furthermore, because our groups were formed by clustering subjects with similar comorbidity profiles within our specific sample, there exist other symptom combinations whose analysis could lead to a more precise determination of comorbidity influence but are absent in our study, rendering our current analysis a preliminary approach. Additionally, the unequal gender distribution across our groups—also determined by the prevalence of the studied comorbidities—precludes us from determining the effect of this parameter on our analysis. Future studies that account for these aspects should be conducted to refine and strengthen our interpretation.

The main limitation of our study is the size of the groups analyzed. Despite the total number of participants exceeding 300, the number of subjects within each specific group was relatively small. This limitation is difficult to overcome, as the study design depended on participants presenting the same set of comorbidities. Nevertheless, statistical analyses revealed significant differences with meaningful effect sizes, suggesting a substantial impact of comorbidities on the measured variables. Future research involving larger groups and a broader range of cognitive tests will provide a deeper understanding of the implications of comorbidities for cognitive performance and brain function.

While our findings successfully highlight cognitive and neurophysiological differences associated with the presence of various comorbidities, it is not possible to directly generalize these results to the broader clinical population presenting with similar symptoms; thus, immediate clinical application is not yet feasible. Furthermore, although we evaluated the presence of symptoms from multiple conditions, our study did not encompass several other psychiatric and medical conditions that could also influence cognitive function and brain electrical activity. However, this methodological limitation does not diminish the value of our study, as its primary aim was conceptual: to emphasize the necessity of systematically assessing comorbidities to enhance the accuracy of patient diagnosis and the efficacy of treatment selection. In this specific context, our results strongly suggest the significance of exploring a patient’s complete comorbidity profile.

To understand the objective of this study, it is important to recognize that variations in brain function give rise to symptoms that, according to clinical guidance documents such as the DSM-5, are interpreted as indicative of specific psychiatric conditions. This classification is essential for patient diagnosis and treatment and has evolved across different editions of the manual in an effort to provide descriptions that clearly correspond to distinct disorders, facilitating more efficient patient identification and the implementation of effective interventions. However, it should be noted that the DSM-5 remains an attempt to categorize symptoms resulting from physiological changes in brain function into discrete clinical entities. The limitations of the DSM-5 have been previously discussed, highlighting aspects that are often overlooked, such as comorbidities and within-disorder symptom heterogeneity, which can impact patient diagnosis and treatment [12,13]. The simultaneous presence of symptoms from different psychiatric disorders does not imply a crossover between conditions, but rather reflects a specific constellation of symptoms arising from physiological changes that do not fit neatly into a single disorder, yet incorporate features currently assigned to multiple disorders.

Pharmacological treatments are commonly employed in the management of psychiatric conditions. Prescribed medications are specifically designed to influence the activity of various neurotransmitter systems to regulate brain function, ultimately leading to a reduction in symptomatology. Theoretically, if the variations in brain function among all patients with the same condition were similar, the medication should be expected to exert a uniform therapeutic effect. However, this uniformity is rarely observed in clinical practice. For instance, some ADHD patients demonstrate a lack of response to both stimulant and non-stimulant treatments; similarly, the presence and intensity of adverse reactions to medication show considerable variability [69]. This variability may stem from several factors, including the specific etiology of ADHD in each case. Furthermore, it is plausible that patients present with undocumented comorbidities—since comprehensive assessment batteries are not routinely included in all clinical studies, and subjects with comorbidities are frequently excluded from analyses—which could also be profoundly influencing underlying brain function and, consequently, treatment efficacy.

Our results do not allow us to assess any neurochemical effects, as they focus solely on brain electrical activity and cognitive performance associated with the presence of comorbidities. Nevertheless, they suggest that comorbidities may be linked to changes in brain electrical activity. Since brain electrical activity arises from the activation of neurochemical synapses, it is reasonable to assume that differences in electrical activity could reflect differences in neurotransmitter system function. While this relationship is not yet fully understood, several studies have examined the connection between brain electrical activity and neurotransmitter systems. For example, a study in Alzheimer’s patients found an association between decreased occipital theta (6–7 Hz) rhythm and low concentrations of adrenaline in the thalamus (post-mortem) [70]. Another study in Parkinson’s disease (PD) patients, linking brain electrical activity with fluorodopa uptake (via PET/CT), reported that increased frontal delta and theta activity was associated with decreased dopaminergic activity in the caudate nucleus and putamen [71]. A subsequent study using quantitative EEG and PET in PD patients found an inverse correlation between theta activity and the density of α2 adrenergic receptors in the frontal cortex [72]. Although these studies do not provide direct evidence that the changes in theta/beta ratio observed in our study correspond to alterations in the neurochemical environment due to comorbidities, they do support a link between brain electrical activity and neurotransmitter function, including dopaminergic and noradrenergic systems. Furthermore, the differential response to pharmacological treatments reported among psychiatric patients indicates that the effects of a drug on neurochemical and electrical activity may vary between individuals. Taken together, this reasoning allows us to speculate—hypothetically—that changes in brain activity associated with comorbidities could alter the neurochemical environment in which a pharmacological treatment acts, highlighting the importance of considering comorbidities in treatment selection. The impact of comorbidities on diagnostic accuracy and treatment selection has been previously proposed [12,13]. However, future, more extensive studies are needed to fully support this theory.

To effectively overcome the challenge of heterogeneous treatment response, further research must be conducted focusing on the neurophysiological variations present in subjects with comorbidities. The focus of these subsequent studies should center on the most common comorbidity profiles for specific conditions and involve the inclusion of a larger battery of cognitive tests, which would enable the characterization of more specific neurocognitive profiles. This approach would serve as a critical starting point for a more precise identification of the optimal treatment required for each profile. This goal does not necessarily entail adding a new medication for every symptom; rather, it suggests developing an individualized combination of pharmacological and/or non-pharmacological interventions that is most suitable for the patient’s unique neurophysiological presentation.

## 5. Conclusions

The presence of comorbid alcohol use disorders affects cognitive performance and brain electrical activity in individuals who already exhibit symptoms of depression, suicide risk, and ADHD. Hypothetically, these changes could also influence the neurochemical environment on which pharmacological treatments act, contributing to variations in therapeutic effects and adverse reactions among different patients. While our results should be considered preliminary due to the study’s limitations, further research will enable a more precise characterization of the neurophysiological and neuropsychological profiles associated with the most common comorbidities, facilitating clinical application through improved identification of the most effective therapeutic approach for each case.

## Data Availability

The data presented in this study are available on request from the corresponding author. The data are not publicly available due to privacy restrictions.
